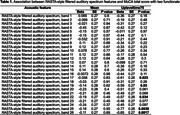# Digital Voice as an Alternative Screening Tool to the Montreal Cognitive Assessment

**DOI:** 10.1002/alz70856_106013

**Published:** 2026-01-08

**Authors:** Hamzah Anan, Amjad Ajam, Abdulrazzaq Qattea, Xavier Serrano, Edward Searls, Kristi Ho, Zexu Li, Alexa Burk, Margaret Low, Owen Tan, Chenglin Lyu, Eric G. Steinberg, Jesse Mez, Michael L Alosco, Katherine A. Gifford, Vijaya B. Kolachalama, Honghuang Lin, Rhoda Au, Huitong Ding

**Affiliations:** ^1^ Alfaisal University, Riyadh, Riyadh, Saudi Arabia; ^2^ Boston University Chobanian & Avedisian School of Medicine, Boston, MA, USA; ^3^ Boston Medical Center, Boston, MA, USA; ^4^ Computing & Data Sciences, Boston University, Boston, MA, USA; ^5^ University of Massachusetts Chan Medical School, Worcester, MA, USA; ^6^ Boston University School of Public Health, Boston, MA, USA

## Abstract

**Background:**

The Montreal Cognitive Assessment (MoCA) is a commonly used screening tool for cognitive impairment. Despite translation into multiple languages to facilitate broader use globally, there are inherent education and cultural biases that result in variations in cognitive screening accuracies. Acoustic voice features are emerging as a more education, language and culturally agnostic indicator of cognitive status, but as a surrogate to the MoCA has not been adequately explored. This pilot study aimed to examine the association between spectral acoustic features extracted by two functionals and MoCA total scores.

**Method:**

We included 80 participants from the Boston University Alzheimer's Disease Research Center, whose responses to a picture‐description task were digitally recorded and administered the MoCA. Using openSMILE, each recording was divided into 20‐ms frames, using a sliding window that advanced by 10 ms for every segment. For each segment, we extracted 26 RASTA‐style filtered auditory spectrum (bands 1‐26) low‐level descriptors (LLD). Two functionals were applied to the 26 LLD to summarize information across each recording: the mean (mean value of LLD for all segments in each recording) and “upleveltime75” (percentage of time the signal exceeds 75% of the feature range above the minimum). Linear regression models were used to assess the association of these acoustic features with MoCA total scores, adjusting for age, sex, and education.

**Result:**

Participant demographics included age: mean 68.7, SD 9.27 years; 81.25% college graduate or higher; 60.0% women. Among these participants, 29 were cognitively impaired. The mean MoCA score was 26.49 (SD = 2.54). Among the 26 upleveltime75 functional‐based spectral features, 5 showed significant negative associations with MoCA. The strongest effect was observed for band 25 (beta = –0.77, SE = 0.26, *p* =  0.0038). In contrast, no significant associations were observed for the acoustic features generated by the mean functional.

**Conclusion:**

These results suggest that digital voice contains cognitive‐related signals and show promise as a potential globally appropriate cognitive screening tool given the ease in which it can be collected and the availability of automated open‐source tools for analysis. Further exploration analyzing voice recordings across different languages and cultural/education strata are warranted.